# The Agreement between Parent-Reported and Directly Measured Child Language and Parenting Behaviors

**DOI:** 10.3389/fpsyg.2016.01710

**Published:** 2016-11-11

**Authors:** Shannon K. Bennetts, Fiona K. Mensah, Elizabeth M. Westrupp, Naomi J. Hackworth, Sheena Reilly

**Affiliations:** ^1^Department of Paediatrics, The University of MelbourneParkville, VIC, Australia; ^2^Murdoch Childrens Research InstituteParkville, VIC, Australia; ^3^Judith Lumley Centre, La Trobe UniversityMelbourne, VIC, Australia; ^4^The Royal Children’s HospitalParkville, VIC, Australia; ^5^Menzies Health Institute Queensland, Griffith UniversityGold Coast, QLD, Australia

**Keywords:** agreement, bias, Bland–Altman Method, Reduced Major Axis regression, measurement, parent-report, child language, parenting

## Abstract

Parenting behaviors are commonly targeted in early interventions to improve children’s language development. Accurate measurement of both parenting behaviors and children’s language outcomes is thus crucial for sensitive assessment of intervention outcomes. To date, only a small number of studies have compared parent-reported and directly measured behaviors, and these have been hampered by small sample sizes and inaccurate statistical techniques, such as correlations. The Bland–Altman Method and Reduced Major Axis regression represent more reliable alternatives because they allow us to quantify fixed and proportional bias between measures. In this study, we draw on data from two Australian early childhood cohorts (*N* = 201 parents and slow-to-talk toddlers aged 24 months; and *N* = 218 parents and children aged 6–36 months experiencing social adversity) to (1) examine agreement and quantify bias between parent-reported and direct measures, and (2) to determine socio-demographic predictors of the differences between parent-reported and direct measures. Measures of child language and parenting behaviors were collected from parents and their children. Our findings support the utility of the Bland–Altman Method and Reduced Major Axis regression in comparing measurement methods. Results indicated stronger agreement between parent-reported and directly measured child language, and poorer agreement between measures of parenting behaviors. Child age was associated with difference scores for child language; however, the direction varied for each cohort. Parents who rated their child’s temperament as more difficult tended to report lower language scores on the parent questionnaire, compared to the directly measured scores. Older parents tended to report lower parenting responsiveness on the parent questionnaire, compared to directly measured scores. Finally, speaking a language other than English was associated with less responsive parenting behaviors on the videotaped observation compared to the parent questionnaire. Variation in patterns of agreement across the distribution of scores highlighted the importance of assessing agreement comprehensively, providing strong evidence that simple correlations are grossly insufficient for method comparisons. We discuss implications for researchers and clinicians, including guidance for measurement selection, and the potential to reduce financial and time-related expenses and improve data quality. Further research is required to determine whether findings described here are reflected in more representative populations.

## Introduction

The success of early intervention programs relies on accurate and sensitive measurement of intervention processes and outcomes. It is surprising then, that research comparing agreement between different types of measurement methods has been extremely limited. There has been increasing attention over the past decade on early intervention programs targeting parenting behaviors in order to improve children’s language outcomes. Language delay affects around one in five children at age four (e.g., Reilly et al., 2010) and persistent difficulties can impact upon future academic success, employment prospects and socio-emotional functioning (Campbell and Ramey, 1994; Stothard et al., 1998). Parenting characterized by warm, positive and responsive interactions can facilitate language development in the early years (Sim, 2012; Cartmill et al., 2013), serving as a buffer against the above-mentioned risks. Understanding how to identify language concerns and intervene early relies upon accurate and reliable measurement. This paper uses existing data from two early childhood cohorts to examine agreement between parent-reported and directly measured child language and parenting behaviors, and the socio-demographic predictors of the difference between measures.

Research focused on understanding complex child developmental and family processes requires highly sensitive assessment tools. Two primary options for researchers seeking to quantify constructs related to child language and parenting behaviors are parent-reported measures and direct (observational or standardized) measures. Both possess notable strengths and limitations, yet there is a lack of comparable data to help researchers identify the circumstances in which one or both methods should be employed. Parents are uniquely positioned to report on their children’s behavior retrospectively and across multiple settings (Gartstein and Marmion, 2008). Parent-reported data is relatively straightforward and inexpensive to collect and analyze (Hawes and Dadds, 2006), making it an appealing measurement approach in large-scale trials where time and cost are significant considerations. However, parents’ unique set of experiences, opinions and attitudes (both explicit and implicit) can contribute to response bias. For example, parents may vary in their interpretation of key terms (Aspland and Gardner, 2003); psychological difficulties can color parents’ perceptions of their children’s behavior (Hayden et al., 2010); or responses can be influenced by social desirability (Law and Roy, 2008). In contrast, direct measures permit the collection of data which is more objective (Wysocki, 2015). For this reason, direct measures are often considered the “gold standard” for assessing both parenting behaviors (Hawes and Dadds, 2006) and child language (Sachse and Von Suchodoletz, 2008). However, collection of such measures requires considerable time and financial resources (Gardner, 2000), and generalizability to other time points and settings has been questioned (Gardner, 1997).

Myriad factors can affect observed behavior or parent-reported responses. Direct measures can be influenced by the presence of the observer or assessor, illness, tiredness, or distractions. Parent-reported measures may be biased by factors associated with a parent’s background. Parents from low socio-economic backgrounds (e.g., low income, low education) have been shown to over- or under-estimate children’s vocabulary on the Communicative Development Inventory (Roberts et al., 1999; Feldman et al., 2000), suggesting that caution in interpretation is required. Furthermore, acquiescence or “yea-saying” (i.e., the tendency to agree with items irrespective of their content) may be a particularly important consideration when administering parent-reported measures with socially disadvantaged populations (Meisenberg and Williams, 2008). It has also been suggested that less educated parents may be less able than well-educated parents to discriminate between expressive and receptive items on a vocabulary checklist, thus providing an inflated estimate of their child’s language abilities (Reese and Read, 2000). Child characteristics such as temperament and gender have similarly been shown to affect parent responses or parent behaviors (Hayden et al., 2010; Olino et al., 2013).

In light of these relative strengths and limitations of parent-reported and direct measures, it is important to establish the extent to which these measurement methods concur and for whom. This information will allow researchers to make more informed decisions about the most appropriate and cost-effective measurement option, given the specific context of their study and finite study resources. For example, given evidence of strong agreement between parent-reported and direct measures, researchers may opt to administer only parent-report; whereas evidence suggesting weak agreement may require researchers to administer multiple methods or only the agreed “gold standard” method.

Few studies have investigated agreement between parent-reported and directly measured behaviors. Of those that have, two primary limitations can be identified. Firstly, these studies tend to employ small sample sizes (in the range of *N* = 50–70). While understandable given the expense associated with using direct measures, small samples reduce the power of the study to identify the limits of agreement with precision. Secondly, these studies typically employ correlational analyses to quantify agreement between measures. For example, moderate correlations have been reported between parent-reported and directly measured child language (e.g., Ring and Fenson, 2000; Sachse and Von Suchodoletz, 2008) and weak to negligible correlations between parent-reported and directly measured parenting behaviors (e.g., Arney, 2004). The use of correlations is problematic because correlations provide a single figure representing the strength of the association between two related variables; they do not assess agreement (Eadie et al., 2014). That is, correlations do not allow for differences in agreement to be examined *across* the spectrum, and they do not account for bias which may be present between two measures, including fixed bias (i.e., bias which is constant across the distribution) or proportional bias (i.e., bias which varies proportionally across the distribution) (see Bland and Altman, 1986; Carstensen, 2010; Bennetts et al., in press). We agree with Stolarova et al. (2014) that greater awareness of the difference between agreement and correlation will lead to the use of more appropriate statistical methods.

Methods such as the Bland–Altman Method (Bland and Altman, 1986) or Reduced Major Axis (RMA) regression (Ludbrook, 2010) represent appropriate alternatives for assessing agreement, allowing researchers to quantify fixed and proportional bias, respectively. These techniques are commonly used for method comparisons in fields such as medicine and chemistry, but are seldom applied in psychology due to a lack of awareness and paucity of literature in the field (Miles and Banyard, 2007). The Bland-Altman Method involves plotting the mean of two measures against the difference between two measures (Altman and Bland, 1983). This provides a visual means of examining the variation in agreement across the spectrum of scores. RMA regression is particularly helpful for identifying proportional bias between measures (Ludbrook, 1997). Execution of this technique involves minimizing the sum of the vertical and horizontal residuals. RMA is suitable for contexts in which measurement error is present in both *x* and *y*, as would be expected in the current study.

This study used data from two cohorts of parents and their children aged 6–36 months to: (1) quantify the agreement between parent-reported and directly measured child language and parenting behaviors, and to (2) determine the association between a range of socio-demographic factors and the difference between parent-reported and direct measures.

## Materials and Methods

### Participants

Data were drawn from two randomized controlled trials of early childhood parenting interventions; (1) a community-based sample of parent–child dyads participating in the Language for Learning program for slow-to-talk toddlers aged 24 months (*N* = 201), and (2) parent–child dyads participating in the Early Home Learning Study; an evaluation of a community-based program to support disadvantaged parents to provide a rich home learning environment for their children aged 6–36 months (*N* = 218). Parents and children completed a suite of assessments, including parent-reported and direct measures of child language and parenting behaviors.

Language for Learning participants were recruited by maternal and child health nurses in three local government areas in Victoria, Australia. All children residing in these areas were recruited at 12 months of age. Children were excluded if there was a known cognitive delay, a major medical condition, or if parents were unable to complete written questionnaires. At child age 18 months, parents completed the Sure Start Language Measure. Children falling below the 20th percentile were invited to participate in the current study of slow-to-talk toddlers.

Early Home Learning Study participants were recruited by child and family service workers and maternal and child health nurses within twenty local government areas in Victoria, Australia. Eligibility criteria included: living within the geographical boundaries of a trial locality; having at least one child aged 6–36 months; and evidence of at least one risk indicator for social disadvantage including: low family income; receipt of government benefits (e.g., Health Care Card for low income families); single, socially isolated or young parent (<25 years); and culturally and linguistically diverse background. Parents were not eligible if they were aged less than 18 years, did not speak English, or were receiving intensive support or child protection services.

### Measures

A summary of parent-reported and direct measures administered for each cohort is provided in **Table 1**. Both cohorts completed parent-reported and direct measures of child language. Direct measures included a standardized language assessment for the Language for Learning cohort, and a videotaped observation for the Early Home Learning Study cohort. Participants in the Early Home Learning Study also completed a videotaped observation of parent–child interaction, as well as parent-reported measures of parenting behaviors.

**Table 1:** Parent-reported and direct measures.

	Parent-reported measures	Direct measures
Child language	• Sure Start Language Measure (SSLM)^a^ • Ages and Stages Questionnaire (ASQ)^a,b^ communication subscale • Macarthur-Bates Communicative Development Inventory (Short-Form, CDI)^b^	• Preschool Language Scale (PLS-4)^a^ • Early Communication Indicator (ECI)^b^
Parenting behaviors	• Parental Verbal Responsivity (PVR)^b^ • Home Activities with Child (HAC)^b^	• Indicator of Parent-Child Interactions (IPCI)^b^

^a^*Language for Learning*; ^b^*Early Home Learning Study*.

#### Parent-Reported Measures

##### MacArthur-Bates Communicative Development Inventories

The CDI is a brief, reliable and commonly used measure of children’s language skills (Fenson et al., 2000). One of three versions was used depending on the child’s age in months. The CDI Short-Form Level I was used for children up to 18 months, consisting of an 89-word list, resulting in a total score from 0 to 89. Parents were asked to indicate if their child “understands” or “understands and says” each word. Parents of children aged 19–30 months completed the Short-Form Level II. Parents were asked to report whether their child ‘says’ 100 listed words resulting in a total score from 0 to 100 for word production and a single item assessing word combinations. Parents of children aged 31 months and above completed the CDI III, consisting of a 100-word vocabulary checklist, 12 sentence pairs to evaluate complexity of language use, and 12 yes/no items assessing language comprehension, resulting in a total score from 0 to 124. Minor changes in word items were made for the Australian context, in-line with other Australian studies (Reilly et al., 2009; Skeat et al., 2010). Scores were standardized for each of the three age-appropriate versions.

##### Sure Start Language Measure

Children’s expressive vocabulary was assessed with the Sure Start Language Measure (SSLM) 100-word checklist (Roy et al., 2005). The SSLM was developed based on the commonly used MacArthur-Bates Communicative Development Inventory, with some items adjusted for the United Kingdom, rather than American context. Parents were asked to indicate whether their child could say 100 words, (e.g., “meow,” “finish” or “happy”) and whether their child was combining words “not yet,” “sometimes” or “often” to produce a total score out of 100.

##### Ages and Stages Questionnaire (ASQ-3) communication subscale

The ASQ allows for developmental and social-emotional screening of children, aged between 1 and 66 months (Squires et al., 2009). Questionnaires comprise five sub-scales: communication, gross motor, fine motor, problem solving, and personal-social, with six items in each subscale, plus an additional 8 open-ended questions addressing overall child development. Only the communication subscale is reported here. Parents were asked to indicate whether their child performs a specific activity using three response categories: ‘yes,’ ‘sometimes’ or ‘not yet’ across six items, each scored as 10, 5, or 0 for ‘yes,’ ‘sometimes’ or ‘not yet’ respectively (e.g., “Does your child correctly use at least two words like “me,” “I,” “mine” and “you”?). Scores were summed to give a total score ranging from 0 to 60. Higher scores indicated stronger communicative abilities. Fourteen age-appropriate versions were administered; therefore scores were standardized within age bands to derive *z*-scores.

##### Parental Verbal Responsivity

The four-item PVR subscale from the StimQ-Toddler (Dreyer et al., 1996) measures how verbally responsive the parent is in interactions with their child on a dichotomous “yes”/”no” scale. To detect greater variability, an alternative 4-point Likert scale was used, where 1 = not at all and 4 = every day (e.g., “I talk about the day while my child is eating”). Scores were summed to produce a total score between 4 and 16, with higher scores indicating greater Parental Verbal Responsivity.

##### Home Activities with Child

The five-item “Home Activities with Child” scale (Nicholson et al., 2008) assessed the frequency with which parents engage in developmentally important activities with their child in a typical week. The scale is administered on 4-point Likert scale, where 1 = not at all, and 4 = every day (e.g., “How often do you involve your child in everyday activities at home, such as cooking or caring for pets?”). Item scores were summed to produce a total score between 5 and 20, with higher scores indicating greater frequency of home activities between the parent and child.

#### Direct Measures

##### Preschool Language Scale, Fourth Edition

The PLS-4 (Zimmerman et al., 2002a) is a standardized and norm-referenced instrument to evaluate children’s receptive and expressive language skills from birth to 6 years and 11 months. This assessment can be used as a screening tool for a range of developmental delays such as problems with language, articulation, connected speech, social communication skills, stuttering, or voice disorders. Although this measure is normed on a US, rather than Australian sample, (*n* = 1564) (Zimmerman et al., 2009), it is one of the most widely used, directly assessed, standardized tools for assessing language ability in very young children. The PLS-4 has been used in other Australian studies with young children (e.g., Ching et al., 2013). This study reports only on the PLS standard score for expressive language.

##### Early Communication Indicator

The ECI (Carta et al., 2010) aims to assess early communicative development of children aged 6–36 months across four key domains: vocalizations; single words; multiple words; and gestures. Parents were asked to play with their child with a standardized set of toys for 6 min while being videotaped. Accredited expert coders scored video data according to standardized protocols. Frequencies for each of the four domains were recorded at 1-min intervals. A total communication composite score was generated by weighting single words by two and multiple words by three, before summing all four domain scores. Inter-rater agreement on 20% of observations independently coded by both assessors was 93.9%, consistent with previously reported figures (Greenwood et al., 2010). Families from a non-English speaking background were not instructed to speak English. Rather, all families were asked to “do what they normally do.” Videos featuring families who spoke a language other than English could not be coded due to the need to employ interpreters; only families who chose to interact in English are included in this analysis.

##### Indicator of Parent–Child Interactions

The IPCI (Carta et al., 2010) was used to quantify the frequency of specific parent and child behaviors during a set of four common early childhood activities: free play (4 min); looking at books (2 min); distraction (2 min); and getting dressed (2 min). The distraction task required parents to keep their child on a small blanket without the child touching a small musical device which was placed within reach. This activity was not administered to children less than 12 months of age. The activities are designed to elicit natural interactions which would typically occur between the parent and child. Activities were videotaped, resulting in a total of 8–10 min’ footage. Accredited expert coders scored video data according to standardized protocols by counting the frequency of interactions for each activity across six parent domains: conveys acceptance and warmth; uses descriptive language; follows child’s lead; maintains or extends child’s focus; uses criticism or harsh voice; uses restrictions or intrusions. For each activity, a relative frequency was allocated to each domain based on a 4-point scale where 0 = never; 1 = rarely; 2 = sometimes or inconsistently; 3 = often or consistently. After each activity was rated, a domain percentage score was calculated by summing all activity scores and dividing by the total number of possible points for that domain. This study reports on the total positive caregiver score only, which captures the frequency of responsive parenting behaviors occurring during the videotaped observation. This total score was generated by summing the percentage scores for the first four domains listed above. Inter-rater agreement on 20% of observations independently coded by both assessors was 87.4%, consistent with previously reported figures (Baggett and Carta, 2006). As described above, all families were asked to “do what they normally do.” However, videos featuring families who spoke a language other than English could not be coded due to the need to employ interpreters.

##### Socio-Demographic Factors

Variables available for both cohorts included: parent age, child age, child gender, parent education, household income, household unemployment, language other than English and socio-economic disadvantage. Socio-economic disadvantage was assessed with the Socio-Economic Indexes for Areas Disadvantage indicator (Australian Bureau of Statistics, 2011), which summarizes the economic and social circumstances for people and households in a particular area (*m* = 1000; *SD* = 100). Lower scores indicate greater disadvantage. A single-item indicator of child temperament was included for both cohorts (higher scores indicated more difficult temperament). Additional variables were included in the Early Home Learning Study analysis due to availability of data, and evidence that these factors may affect parent responses or behavior: global parenting self-efficacy, assessed using a single-item indicator (“Overall, as a parent, do you feel that you are …” not very good at being a parent; a person who has some trouble being a parent; an average parent; a better than average parent; a very good parent); psychosocial distress assessed with the K6 (Kessler et al., 2002); and health-related quality of life evaluated with the SF-12 UK version (Jenkinson and Layte, 1997).

### Procedure

*Language for Learning:* Children identified as slow-to-talk at 18 months were assessed at 24 months by a trained research assistant. Researchers visited families at home to collect parent-reported data and to administer a standardized child language assessment. *Early Home Learning Study:* Prior to intervention, trained research assistants videotaped parents and children at home during play activities to examine child language development and parent–child interactions. Parents also completed a brief measure of child language during the visit. A 30-min parent questionnaire was administered via computer-assisted telephone interview.

Ethical approval for the Language for Learning study was granted by the Royal Children’s Hospital Human Research Ethics Committee (EHRC #26028) and The University of Melbourne (#0829736). All parents provided written informed consent. Ethical approval to access existing Language for Learning data for the current study was covered under the Centre for Excellence in Child Language and approved by the Royal Children’s Hospital Human Research Ethics Committee (HREC #32261 B). Ethical approval for the Early Home Learning Study was granted by the Victorian Government Department of Health (HREC 08/10). All parents provided written informed consent. Ethical approval to access existing Early Home Learning Study data for the current study was granted by The University of Melbourne Human Research Ethics Committee (ID 1543863.1).

### Statistical Analyses

All analysis was conducted using Stata/IC Version 13.0 (StataCorp, 2013). Prior to analyses, two fathers were excluded from the *Language for Learning* dataset and nine from the Early Home Learning Study dataset, given that parent gender has been found to contribute to differences in data collection (Olino et al., 2013) and the inclusion of such small numbers of fathers was considered insufficient to identify differences between mothers and fathers. A total of nine measures were examined across the two cohorts. Between these measures, nine comparisons were conducted: six compared parent-reported and directly measured behaviors, and three compared parent-reported and parent-reported behaviors. Histograms of the differences were examined for all nine comparisons, followed by scatterplots with a line of best fit to examine linear association. Both Pearson’s Correlation Coefficients and Spearman Rank Correlation Coefficients were calculated for each comparison. Pearson’s is reported here to enable cross-study comparisons with existing literature, and Spearman’s is also reported to account for non-normality of distributions. The Concordance Correlation Coefficient (CCC) was also computed using the Stata “-concord” command. Developed by Lin (1989) as a measure of agreement, the CCC quantifies the degree to which pairs of observations fall on the 45° line through the origin. It contains a measure of precision using the Pearson’s Correlation Coefficient, as well as a bias correction for accuracy.

*Z*-scores were derived for each of the outcome variables to enable cross-measure comparisons on the same scale. Bland–Altman plots were then generated using the Stata “-concord” command (Cox and Steichen, 2007) for all nine comparisons. This plots the mean of the measures against the difference between the measures, as well as the line of mean difference and the 95% limits of agreement. RMA regression (or “ordinary least products” regression) was conducted using the Stata “–concord” command.

The associations between a range of socio-demographic factors and the difference between *z*-scores were estimated using unadjusted and adjusted linear regression. Difference scores were calculated by subtracting one *z*-score from the other, and these were then used as the outcome variables for the regressions. Unadjusted associations were examined, before the adjusted models were tested. Only variables associated with the outcome at *p* ≤ 0.1 were included in the adjusted models. All continuous variables were screened for evidence of multicollinearity (*r* ≥ 0.70); none were excluded. Factors included in the adjusted models for both cohorts included parent age, child age, child gender, parental education, household income, household unemployment, SEIFA disadvantage score, language other than English and a single-item indicator of child temperament. Additional variables included in the analyses from the Early Home Learning Study dataset were parenting self-efficacy, psychosocial distress, and health-related quality of life. The Stata “-mixed” command was used for this cohort, to account for the cluster-RCT study design and Intraclass Correlation Coefficients were examined.

Finally, quantile regressions were conducted to determine whether the association between the socio-demographic factors and the difference scores varied across the distribution of the difference scores. Associations were examined across the 25th, 50th, and 75th quantiles. Each model was compared to the standard ordinary least squares output and a test for heteroscedasticity was used to determine whether there was evidence against the null hypothesis of constant variance across the quantiles.

### Sample Size

Bland (2004) provides a formula to evaluate the precision of the sample size to accurately assess agreement between measures. Bland advises that the 95% confidence interval around the limits for agreement may be estimated as ±1.96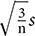 where *s* is the standard deviation of the differences between measurements by the two methods, and *n* is the sample size. Bland recommends that a sample size of 100 is adequate for method comparisons. Applying this formula provides excellent precision for the Language for Learning cohort of *N* = 201 (±0.24 s). For the Early Home Learning Study, direct measures were only available for a subset of the cohort (Early Communication Indicator, *N* = 100; and Indicator of Parent–Child Interactions, *N* = 163) providing adequate precision for comparisons involving these measures (±0.34 s and ±0.27 s, respectively).

## Results

### Sample

Sample characteristics for each study are summarized in **Table 2**. *Language for Learning*: Nearly half of the parents had completed higher education and fewer than one in 10 families spoke a non-English language. There were approximately equal proportions of male and female children, and more than three-quarters of parents were married. Most parents reported earning a mid to high range household income, with one in five reporting a low income. *Early Home Learning Study*: Similar characteristics were observed in terms of education, marital status and child gender compared to families in the Language for Learning study. However, Early Home Learning Study parents were more likely to be younger, to speak a language other than English, and to live in a household without an employed person. Language for Learning participants were on average, less disadvantaged compared with the Australian mean (*m* = 1026.6) and Early Home Learning Study participants were slightly more disadvantaged (*m* = 984.2); however, there was also a large degree of variation in scores (ranges: 888.2–1117.5 and 816.7–1105.9, respectively).

**Table 2:** Sample characteristics for participants in each cohort.

Variable	Language for Learning (*n* = 201)	Early Home Learning Study (*n* = 218)
Parent age, years, mean *(SD)*	35.3 (4.4)	32.6 (5.1)
Child age, months, *mean (SD)*	24.4 (1.1)	16.2 (9.3)
Child female, *n* (*%)*	95 (47.0)	113 (51.8)
Parent marital status n, (%)		
Single/separated/divorced	11 (5.5)	17 (7.8)
Married/de facto	190 (94.5)	201 (92.2)
Household unemployment *n (%)‘*	10 (5.0)	18 (8.3)
Parent education, *n (%)*		
Higher education	93 (46.7)	112 (51.4)
No higher education	106 (53.3)	106 (48.6)
LOTE, 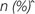	19 (9.5)	46 (21.1)
Household income p/a, *n (%)*^∗^		
<$46,800	38 (19.3)	–
$46,800–$70,200	69 (35.0)	–
>$70,200	90 (45.7)	–
<$36,400	–	26 (12.0)
$36,400–51,999	–	36 (16.6)
≥ $52,000	–	147 (67.7)
SEIFA^#^, *mean (SD)*	1026.6 (54.1)	984.2 (57.9)

*‘Single parent unemployed or both parents unemployed; ˆLanguage other than English; ^∗^Different categories of income were administered for each sample; ^#^Socio-Economic Index for Areas (SEIFA) Disadvantage score is an indicator of relative disadvantage, based on postcode of residence, accounting for low income, low educational attainment and high unemployment. Lower index scores indicate greater disadvantage*.

### Descriptive Statistics

The means, standard deviations and ranges for the parent-reported and directly measured behaviors are presented in **Table 3**. Alpha coefficients indicate excellent internal consistency for the SSLM and Preschool Language Scale, consistent with figures reported elsewhere (Zimmerman et al., 2002b; Roy et al., 2005; Zubrick et al., 2007). There was poorer internal consistency for Parental Verbal Responsivity and the Home Activities with Child scales, which is typically expected for measures with few items (Gliem and Gliem, 2003). Children’s expressive language standard scores on the Preschool Language Scale (*m* = 91.2, *SD* = 12.3) indicate that, on average, children were performing below the 50th percentile. As shown, direct measures were only available for a sub-sample of participants in the Early Home Learning Study cohort. Due to the financial expenses associated with video coding, the data used in this paper represents a sub-sample of a larger dataset; this sub-sample was selected at random.

**Table 3:** Descriptives for parent-reported and directly measured behaviors.

	*M (SD)*	Range	α	*N* Missing from Total Sample *N*
**Child language**				
Sure Start Language Measure^a^	35.0 (22.7)	0 to 98	0.97	7/201
Ages and Stages Questionnaire^a∗^	0 (1)	-2.8 to 1.1	n/a	1/201
Ages and Stages Questionnaire^b∗^	0 (1)	-3.0 to 1.7	n/a	1/218
Communicative Development Inventory^b^	100.4 (9.7)	81.0 to 160.7	n/a	5/218
Preschool Language Scale^a^	91.2 (12.3)	64 to 135	0.86	2/201
Early Communication Indicator^b^	10.1 (7.3)	0.3 to 32.3	n/a	118/218
**Parenting behaviors**				
Parental Verbal Responsivity^b^	12.9 (2.21)	6 to 16	0.40	0/218
Home Activities with Child^b^	17.1 (2.52)	9 to 20	0.49	0/218
Indicator of Parent–Child Interactions^b^	200.1 (55.3)	50 to 370	n/a	55/218

^a^*Language for Learning*; ^b^*Early Home Learning Study; ^∗^Z-Scores were derived to account for different age-appropriate versions.*

### Correlations

The strongest correlations were obtained for comparisons involving two parent-reported measures, with moderate positive associations (see **Table 4**). The strongest correlation for any parent-reported and direct comparison was between the Ages and Stages Questionnaire (communication subscale) and Preschool Language Scale (expressive language), with a moderate, positive correlation. Weaker associations were obtained for the remaining comparisons, with a moderate positive correlation between the Communicative Development Inventory and the Early Communication Indicator, and a weak non-significant correlation between the Ages and Stages Questionnaire and the Early Communication Indicator. Associations between measures of parenting behaviors were much weaker than the child language comparisons, with near-negligible associations between parent-reported and direct measures. In contrast to the Pearson’s and Spearman’s coefficients, which produced similar coefficients for each comparison, the Lin’s CCC produced markedly smaller correlations for several comparisons. This suggests that, although the measures are associated, the level of *agreement* is much poorer. Lin’s correlation line passes through the origin, with a slope of one. Thus, it provides a measure of correspondence between measures, rather than association. As shown in **Table 4**, the Lin’s coefficient for two comparisons was close to zero, yet highly significant. The confidence intervals for these comparisons were very narrow, hence the significant *p*-values.

**Table 4:** Pearson’s (*r*), Spearman’s Rank (*ρ*) and Lin’s Concordance (ρ_c_) correlation coefficients for each of the nine comparisons.

	*r*	*ρ*	ρ_c_
**Child language**			
(1) Ages and Stages Questionnaire vs. Sure Start Language Measure	0.70^∗∗∗^	0.73^∗∗∗^	0.70^∗∗∗^
(2) Ages and Stages Questionnaire vs. Preschool Language Scale	0.61^∗∗∗^	0.59^∗∗∗^	0.61^∗∗∗^
(3) Sure Start Language Measure vs. Preschool Language Measure	0.56^∗∗∗^	0.60^∗∗∗^	0.56^∗∗∗^
(4) Ages and Stages Questionnaire vs. Communicative Development Inventory	0.44^∗∗∗^	0.48^∗∗∗^	0.001^∗∗∗^
(5) Ages and Stages Questionnaire vs. Early Communication Indicator	0.12	0.16	0.01
(6) Communicative Development Inventory vs. Early Communication Indicator	0.32^∗∗^	0.33^∗∗^	0.01^∗∗^
**Parenting behaviors**			
(7) Parental Verbal Responsivity vs. Home Activities with Child	0.45^∗∗∗^	0.45^∗∗∗^	0.17^∗∗∗^
(8) Parental Verbal Responsivity vs. Indicator of Parent–Child Interaction	-0.03	-0.04	0.00
(9) Home Activities with Child vs. Indicator of Parent–Child Interaction	0.06	0.08	0.00

*^∗∗∗^p ≤ 0.001; ^∗∗^p ≤ 0.01; ^∗^p < 0.05.*

### Agreement between Methods

**FIGURE 1:**
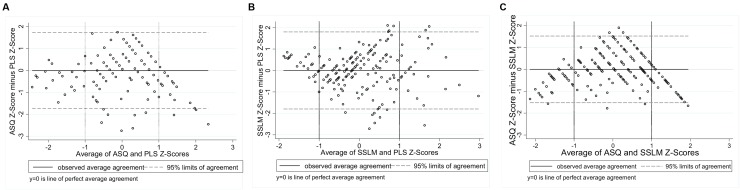
**Bland-Altman Plots: (A)** Ages and Stages Questionnaire and Preschool Language Scale [95% limits of agreement: -1.75 to 1.73]; **(B)** Sure Start Language Measure and Preschool Language Scale [95% limits of agreement: -1.80 to 1.80]; **(C)** Ages and Stages Questionnaire and Sure Start Language Measure [95% limits of agreement: -1.52 to 1.53].

**FIGURE 2:**
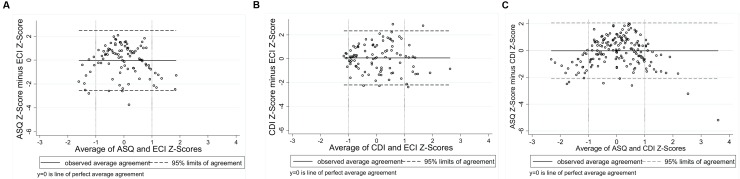
**Bland–Altman Plots: (A)** Ages and Stages Questionnaire and Early Communication Indicator [95% limits of agreement: -2.55 to 2.48]; **(B)** Communicative Development Inventory and Early Communication Indicator [95% limits of agreement: -2.21 to 2.33]; **(C)** Ages and Stages Questionnaire and Communicative Development Inventory [95% limits of agreement: -2.10 to 2.06].

**FIGURE 3:**
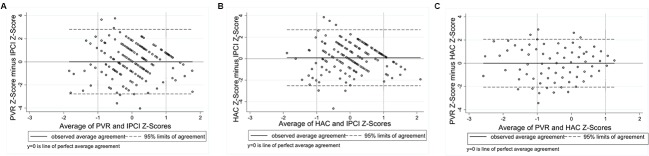
**Bland–Altman Plots: (A)** Parental Verbal Responsivity and Indicator of Parent–Child Interactions [95% limits of agreement: -2.78 to 2.80]; **(B)** Home Activities with Child and Indicator of Parent–Child Interactions [95% limits of agreement: -2.52 to 2.70]; **(C)** Parental Verbal Responsivity and Home Activities with Child [95% limits of agreement: -2.06 to 2.06].

Application of the Bland–Altman Method requires the differences between measures to be approximately normally distributed (Bland and Altman, 2003). Histograms of the differences showed approximate normality, with slight negative skewness evident for the ASQ-ECI and ASQ-CDI, and positive skewness for the PVR-HAC. The association between each of the nine comparisons was examined using scatterplots with a fitted line of equality. Scatterplots suggested a positive, approximately linear relationship whereby higher scores on one measure correspond with increasing scores on the other measure. Exceptions were the PVR-IPCI and HAC-IPCI scatterplots, which did not provide evidence of a linear association. Bland–Altman Plots were generated for each of the nine comparisons (**Figures 1**–**3**). In each plot, the solid horizontal line represents the mean difference between the measures and the dotted lines represent the ‘limits of agreement’ within which 95% of data points lie. The overall bias (mean difference) was close to zero for most comparisons, reflecting the scaling of the measures to *z*-scores; we therefore focus on the limits of agreement and patterns of agreement across the range of scores.

#### Child Language

**Figure 1** shows the three Language for Learning comparisons. Plot A shows the agreement between the parent-reported ASQ and the standardized language measure, PLS. The points are widely dispersed around the mid-section, indicating poorer agreement for children with average language abilities. Points are closest to *y* = 0 at the lower end, indicating the strongest agreement for children with the poorest language abilities. Vertical reference lines at *x* = -1 and *x* = 1 have been included for ease of interpretation. At the upper end of the spectrum (scores > 1 on the *x* axis), points are all below *y* = 0, indicating that the parent-reported ASQ is systematically underestimating children’s language, compared to the direct measure (PLS). The limits of agreement tell us that 95% of the points lie between -1.75 and 1.73 standard deviations. Plot B shows the agreement between the parent-reported SSLM and the standardized language measure, the PLS. At the lower end (scores below *x* = -1) points are more tightly clustered around the line *y* = 0, indicating stronger agreement between these measures for children with poorer language abilities. Agreement then appears to deteriorate across the spectrum, as children’s average language abilities increase. This is shown by the much wider dispersion of points from *x* = 0 and above. The limits of agreement tell us that 95% of the points lie between -1.80 and 1.80 standard deviations. The strongest agreement of all nine comparisons was found for two parent-reported language measures, the ASQ and the SSLM (Plot C), with the narrowest limits of agreement (-1.53 to 1.53 standard deviations). For children with average language abilities (scores between -1 and 1 on the *x*-axis) parents both underestimate and overestimate on the ASQ, compared to the SSLM. For children with poorer language abilities (below -1 on the *x*-axis) and higher average language abilities (above 1 on the *x*–axis), the ASQ produces lower scores than the SSLM.

**Figure 2** shows the three Early Home Learning Study comparisons. Plot A shows the agreement between the parent-reported ASQ and the direct videotaped observation, the ECI. For children with average language abilities, parents are over- and under-estimating their children’s language abilities on the ASQ, compared to scores from the directly measured ECI. For children with poorer language abilities (scores below -1 on the *x*-axis) and stronger language abilities (scores above 1 on the *x*-axis), most points are positioned below the line *y* = 0. This suggests that parents of children with very poor or very strong average language abilities are underestimating on the ASQ, compared to the directly measured ECI. The limits of agreement tell us that 95% of the points lie between -2.55 and 2.48 standard deviations. A different pattern of agreement is evident between the parent-reported CDI and the ECI (Plot B), whereby the strongest agreement occurred for children with the poorest language ability, and agreement progressively deteriorated as children’s language ability improved (95% limits of agreement: -2.21 to 2.33 standard deviations). Not surprisingly, the strongest agreement of the six Early Home Learning Study comparisons was between the two parent-reported measures, the ASQ and the CDI (Plot C) (95% limits of agreement: -2.10 to 2.06 standard deviations). However, the distribution of points suggests that the poorest agreement between the measures is for children with average language abilities (scores between -1 and 1 on the *x*-axis). For children with poorer average language abilities (scores < -1 on the *x*-axis) and stronger average language abilities (scores > 1 on the *x*-axis), the ASQ is underestimating, compared to the SSLM.

#### Parenting Behaviors

**Figure 3** shows poorer agreement between measures of parenting behaviors compared to the child language measures. Plot A presents agreement between the parent-reported PVR and the direct videotaped observation, the IPCI. The more dispersed scatter of points around the mid-section reveals that the poorest agreement is for parents of average responsiveness (95% limits of agreement: -2.78 to 2.80 standard deviations). Parents with poorer average responsiveness (scores < -1 on the *x*-axis) and parents with stronger average responsiveness (scores > 1 on the *x*-axis) tend to underestimate their responsiveness on the PVR, compared to scores on the IPCI. A similar pattern can be seen between the parent-reported HAC and the IPCI (Plot B), with slightly stronger agreement indicated by narrower 95% limits of agreement (-2.52 to 2.70 standard deviations). As shown with the child language comparisons, the strongest agreement between measures of parenting behaviors was between the two parent-reported measures, the PVR and the HAC (Plot C), whereby 95% of the points lie between -2.06 and 2.06 standard deviations. The horizontal scatter of points indicates that the bias between these measures is relatively fixed across the distribution of scores.

### Identification of Proportional Bias

**FIGURE 4:**
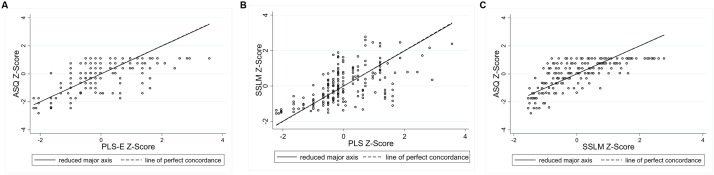
**RMA plots: (A)** Ages and Stages Questionnaire and Preschool Language Scale, Slope = 0.996; Intercept = -0.009; **(B)** Sure Start Language Measure and Preschool Language Scale, Slope = 0.990; Intercept = -0.001; **(C)** Ages and Stages Questionnaire and Sure Start Language Measure, Slope = 1.005; Intercept = 0.002.

**FIGURE 5:**
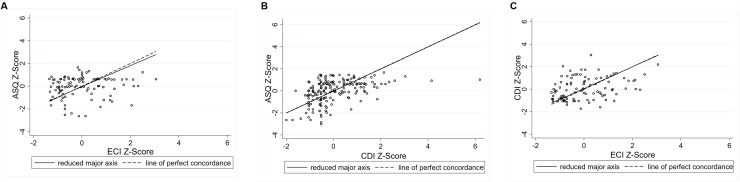
**RMA plots: (A)** Ages and Stages Questionnaire and Early Communication Indicator, Slope = 0.924; Intercept = -0.036; **(B)** Ages and Stages Questionnaire and Communicative Development Inventory, Slope = 1.000; Intercept = -0.020; **(C)** Communicative Development Inventory and Early Communication Indicator, Slope = 0.967; Intercept = 0.064.

**FIGURE 6:**
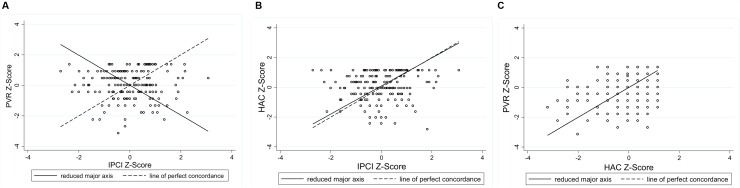
**RMA plots: (A)** Parental Verbal Responsivity and Indicator of Parent–Child Interactions, Slope = -0.98; Intercept = 0.012; **(B)** Home Activities with Child and Indicator of Parent–Child Interactions, Slope = 0.94; Intercept = 0.09; **(C)** Parental Verbal Responsivity and Home Activities with Child, Slope = 1.00; Intercept = 0.00.

**Figures 4–6** present the RMA regression plots to identify the presence of proportional bias. As shown in **Figure 4**, the Language for Learning language measures show very minimal proportional bias, evidenced by the slopes which are close to one and the intercepts which are close to zero. The three Early Home Learning Study child language comparisons also show minimal proportional bias; however, **Figure 5A** shows a degree of bias between the parent-reported ASQ and the directly measured videotaped observation, the ECI, indicated by the slight divergence of lines. **Figure 6** shows the three parenting behavior comparisons. Substantial proportional bias is evident between the parent-reported PVR and the directly measured videotaped observation, the IPCI (**Figure 6A**). This is shown by the strong divergence of lines in the plot. The slope of around -1 indicates that for lower PVR scores, IPCI scores are relatively higher, and for lower IPCI scores, PVR scores are relatively higher. Only slight proportional bias can be seen between the parent-reported HAC and the IPCI (**Figure 6B**). **Figure 6C** indicates the absence of proportional bias between the parent-reported PVR and the parent-reported HAC.

### Socio-Demographic Factors and Agreement

The results of the adjusted linear regressions are presented in **Table 5** for the Language for Learning cohort and **Tables 6** and **7** for the Early Home Learning cohort (See Supplementary Tables for unadjusted models). Non-significant variables at the unadjusted level (*p* > 0.1) were excluded from the adjusted analyses. The outcome variables in all regression analyses are difference scores, calculated by subtracting one *z*-score from another. The intraclass correlations from the multilevel mixed-effects linear regression for each outcome measure (Early Home Learning Study cohort) ranged from 0.00 to 0.22. This reflected the cluster randomized controlled trial design and was accounted for in the regression models.

**Table 5:** Adjusted analysis for Language for Learning difference scores and socio-demographic factors.

	ASQ vs. PLS-E			SSLM vs. PLS-E			ASQ vs. SSLM		
	Coefficient	*p*	95% CI	Coefficient	*p*	95% CI	Coefficient	*p*	95% CI
Parent age (years)	^∗^	^∗^	^∗^	^∗^	^∗^	^∗^	0.03	0.05	0.00, 0.05
Child age (months)	0.23	<0.001	0.12, 0.34	0.35	<0.001	0.23, 0.46	-0.13	0.02	-0.24, -0.02
Child gender (female)	^∗^	^∗^	^∗^	0.18	0.14	-0.06, 0.42	^∗^	^∗^	^∗^
Single parent	^∗^	^∗^	^∗^	0.37	0.24	-0.24, 0.98	^∗^	^∗^	^∗^
Household unemployment	^∗^	^∗^	^∗^	^∗^	^∗^	^∗^	-0.20	0.50	-0.79, 0.38
No higher education	^∗^	^∗^	^∗^	^∗^	^∗^	^∗^	^∗^	^∗^	^∗^
**Income**									
Low vs. mid	^∗^	^∗^	^∗^	-0.22	0.25	-0.59, 0.16	0.27	0.12	-0.07, 0.61
Low vs. high	^∗^	^∗^	^∗^	-0.33	0.08	-0.70, 0.04	0.38	0.02	0.06, 0.71
SEIFA/100 (less disadvantage)	-0.23	0.05	-0.46, 0.00	-0.15	0.22	-0.39, 0.09	^∗^	^∗^	^∗^
LOTE	^∗^	^∗^	^∗^	^∗^	^∗^	^∗^	0.29	0.15	-0.11, 0.69
Difficult child temperament	^∗^	^∗^	^∗^	^∗^	^∗^	^∗^	-0.18	0.02	-0.33, -0.03
	*R*^2^ = 0.09			*R*^2^ = 0.19			*R*^2^= 0.10		

*^∗^Excluded due to p > 0.1 at univariate level; LOTE, Language other than English. ASQ = Ages and Stages Questionnaire, communication subscale; PLS-E = Preschool Language Scale, expressive language score; SSLM = Sure Start Language Measure.*

**Table 6:** Adjusted analysis for the Early Home Learning Study difference scores and socio-demographic factors (child language measures).

	ASQ vs. ECI			CDI vs. ECI			ASQ vs. CDI		
	Coefficient	*p*	95% CI	Coefficient	*p*	95% CI	Coefficient	*p*	95% CI
Parent age (years)	^∗^	^∗^	^∗^	^∗^	^∗^	^∗^	^∗^	^∗^	^∗^
Child age (months)	-0.09	<0.001	-0.12, -0.06	-0.07	<0.001	-0.10, -0.04	^∗^	^∗^	^∗^
Child gender (female)	0.24	0.24	-0.16, 0.64	^∗^	^∗^	^∗^	^∗^	^∗^	^∗^
Single parent	^∗^	^∗^	^∗^	^∗^	^∗^	^∗^	^∗^	^∗^	^∗^
Household unemployment	^∗^	^∗^	^∗^	^∗^	^∗^	^∗^	^∗^	^∗^	^∗^
No higher education	^∗^	^∗^	^∗^	^∗^	^∗^	^∗^	^∗^	^∗^	^∗^
Income									
Low vs. mid	^∗^	^∗^	^∗^	^∗^	^∗^	^∗^	^∗^	^∗^	^∗^
Low vs. high	^∗^	^∗^	^∗^	^∗^	^∗^	^∗^	^∗^	^∗^	^∗^
SEIFA/100 (less disadvantage)	^∗^	^∗^	^∗^	^∗^	^∗^	^∗^	^∗^	^∗^	^∗^
LOTE	^∗^	^∗^	^∗^	^∗^	^∗^	^∗^	0.31	0.08	-0.04, 0.66
Difficult child temperament	-0.50	0.05	-0.99, -0.01	-0.51	0.02	-0.95, -0.07	^∗^	^∗^	^∗^
High parenting self-efficacy	0.06	0.63	-0.18, 0.30	^∗^	^∗^	^∗^	^∗^	^∗^	^∗^
Poor health-related quality of life	-0.07	0.54	-0.29, 0.15	-0.10	0.34	-0.31, 0.11	^∗^	^∗^	^∗^
Greater psychological distress	^∗^	^∗^	^∗^	^∗^	^∗^	^∗^	^∗^	^∗^	^∗^
	*R*^2^= 0.40			*R*^2^= 0.33			*R*^2^= 0.01		

*^∗^Excluded due to p > 0.1 at univariate level; LOTE, Language other than English. ASQ = Ages and Stages Questionnaire, communication subscale; ECI = Early Communication Indicator; CDI = Communicative Development Inventory*.

**Table 7:** Adjusted analysis for the Early Home Learning Study difference scores and socio-demographic factors (parenting behavior measures).

	PVR vs. IPCI			HAC vs. IPCI			PVR vs HAC		
	Coefficient	*p*	95% CI	Coefficient	*p*	95% CI	Coefficient	*p*	95% CI
Parent age (years)	-0.04	0.07	-0.08, 0.00	-0.04	0.02	-0.08, 0.00	^∗^	^∗^	^∗^
Child age (months)	-0.01	0.32	-0.04, 0.01	^∗^	^∗^	^∗^	-0.02	<0.01	-0.04, -0.01
Child gender (female)	^∗^	^∗^	^∗^	-0.47	0.02	-0.85, -0.09	^∗^	^∗^	^∗^
Single parent	^∗^	^∗^	^∗^	^∗^	^∗^	^∗^	^∗^	^∗^	^∗^
Household unemployment	^∗^	^∗^	^∗^	^∗^	^∗^	^∗^	^∗^	^∗^	^∗^
No higher education	0.53	0.01	0.12, 0.93	^∗^	^∗^	^∗^	^∗^	^∗^	^∗^
Income									
Low vs. mid	^∗^	^∗^	^∗^	^∗^	^∗^	^∗^	^∗^	^∗^	^∗^
Low vs. high	^∗^	^∗^	^∗^	^∗^	^∗^	^∗^	^∗^	^∗^	^∗^
SEIFA/100 (less disadvantage)	^∗^	^∗^	^∗^	^∗^	^∗^	^∗^	^∗^	^∗^	^∗^
LOTE	1.23	<0.001	0.66, 1.79	1.09	<0.001	0.56, 1.62	^∗^	^∗^	^∗^
Difficult child temperament	-0.43	0.11	-0.96, 0.10	^∗^	^∗^	^∗^	-0.10	0.59	-0.46, 0.26
Low parenting self-efficacy	0.17	0.16	-0.07, 0.40	^∗^	^∗^	^∗^	^∗^	^∗^	^∗^
Poor health-related quality of life	^∗^	^∗^	^∗^	^∗^	^∗^	^∗^	^∗^	^∗^	^∗^
Greater psychological distress	^∗^	^∗^	^∗^	^∗^	^∗^	^∗^	^∗^	^∗^	^∗^
	*R*^2^= 0.18			*R*^2^= 0.14			*R*^2^= 0.05		

*^∗^Excluded due to p > 0.1 at univariate level; LOTE, Language other than English. PVR = Parental Verbal Responsivity scale; IPCI = Indicator of Parent–Child Interactions, positive caregiver score; HAC = Home Activities with Child scale*.

#### Child Language (Language for Learning Cohort)

Child age was a significant predictor of difference scores for this cohort. Parents of older children tended to report higher child language scores on the parent-reported ASQ and SSLM, compared to scores generated by the directly measured PLS. Older child age was also associated with lower scores on the ASQ, compared with the SSLM. The included predictors explained nearly twice the amount of variance in difference scores for the SSLM and PLS (*R*^2^ = 0.19, 19%), compared to the ASQ and PLS (9%) and the ASQ and SSLM (10%).

#### Child Language (Early Home Learning Study Cohort)

Child age and temperament predicted the difference scores between the parent-reported ASQ and the directly measured ECI, as well as the parent-reported CDI and ECI. For both comparisons, older child age was associated with lower scores on the ASQ and CDI, compared to the ECI. Parents who perceived their child as more difficult also tended to report lower scores on the ASQ and CDI, compared with the ECI. The included predictors explained negligible variance in difference scores between the two parent-reported measures, the ASQ and the CDI (*R*^2^ = 0.01, 1%), but explained substantial variance between the ASQ and ECI (40%) and the CDI and ECI (33%).

#### Parenting Behaviors (Early Home Learning Study Cohort)

The differences between measures of parenting behaviors were associated with parent age and English language status. Parents who spoke a language other than English were more likely than native English speakers to report greater parental responsiveness on a parent questionnaire (PVR or HAC), compared to scores generated from the directly measured IPCI. Older parents were also more likely to report less parent responsiveness on the parent-reported PVR and HAC, compared with scores on the IPCI. The included predictors explained minimal variance in difference scores: PVR and IPCI (*R*^2^ = 0.18, 18%); HAC and IPCI: (14%); PVR and HAC (5%).

### Socio-Demographic Factors across Quantiles of Agreement

Quantile regression analyses provided scant evidence that the association between the socio-demographic factors and the difference scores varied across the distribution of the difference scores. The Breusch–Pagen/Cook–Weisberg test for heteroscedasticity provided non-significant *p*-values for eight of the nine comparisons (Language for Learning: ASQ-PLS, *p* = 0.40; SSLM-PLS, *p* = 0.16; ASQ-SSLM, *p* = 0.31 and Early Home Learning Study: ASQ-ECI, *p* = 0.20; ASQ-CDI, *p* = 0.12; CDI-ECI, *p* = 0.87; PVR-IPCI, *p* = 0.87; PVR-HAC, *p* = 0.11). This suggests that the standard Ordinary Least Squares regression is sufficient for quantifying these associations. However, associations did vary across the distribution of difference scores for the HAC-IPCI comparison (*p* = 0.03). Closer inspection of the HAC-IPCI comparison revealed that income (low vs. mid income), varied across the quantiles of difference (25th quantile: coefficient = 0.27, *p* = 0.68; 50th quantile: coefficient = -0.64, *p* = 0.30; 75th quantile: coefficient = -1.44, *p* = 0.01). That is, participants with a low income were more likely to have a large difference between HAC and IPCI scores, compared to participants with a mid-range income. This finding should be interpreted with caution; given the number of comparisons made, it is potentially attributable to chance.

## Discussion

This is the first study to specifically examine agreement between parent-reported and directly measured behaviors using the Bland–Altman Method and RMA regression. Nine comparisons were conducted using data from two independent Australian cohorts (six child language and three parenting behaviors). Although correlational findings were consistent with extant literature, Bland–Altman plots revealed substantial variation in agreement between parent-reported and directly measured child language and parenting behaviors across the distribution of scores. Agreement was generally stronger for children with poorer or exceptional language abilities, and weaker for children with average language abilities. Particularly for comparisons involving the ASQ, parents tended to underestimate their children’s language abilities, when children’s language was either poor or exceptional. Agreement between measures of parenting behaviors was slightly weaker than child language. Proportional bias between child language measures was minimal, but considerable bias was evident between parent-reported and directly measured parenting behaviors. Differences between child language measures were associated with child age and temperament, and differences between parenting behavior measures were associated with parent age and speaking a language other than English. Findings provide strong evidence that simple correlations are grossly insufficient for method comparisons.

### Child Language

Findings suggest that parent-reported measures are most accurate for children who display either language difficulties or exceptional language abilities. Overall, the strongest agreement was observed for children with the poorest language. This may reflect parental concern and a tendency to more closely observe and monitor child development. Children at either end of the language spectrum may “stand out” from their peers. Reflecting the phenomenon identified in Festinger’s (1954) Social Comparison Theory, parents may rely on social comparisons to inform their decision about their children’s development. Children whose abilities reflect the norm may not generate the same close attention from their parents as children at either end of the spectrum. The variability in child language in the early years is well-established (Ukoumunne et al., 2012), however, it is possible that children at the extreme ends of the spectrum are more stable in their language over time, supporting more accurate measurement for these groups. Whereas parent-reported measures may be sufficient to identify children with very poor or very strong language skills, multiple or gold standard direct measures would be necessary to delineate the language skills of children across the mid ranges of child language.

It should be noted that for comparisons involving the ASQ (**Figures 1A,C** and **2A,C**) parents tended to underestimate children’s language abilities for children with very poor or exceptional language. This may reflect the limited variability captured by the ASQ, given that it is a six-item measure scored on a 3-point scale. For comparisons involving the CDI or the UK version of the CDI (SSLM), a different pattern emerged, whereby agreement with direct measures was stronger for children with poorer language ability and progressively worsened as children’s language abilities strengthened. This may reflect a ceiling effect for this commonly used parent-reported measure of expressive vocabulary, where variation in children with exceptional skills cannot be accurately captured. Indeed, the potential for ceiling effects on the CDI for children aged 27 months and above has been reported elsewhere, particularly for children with more advanced language (Fenson et al., 2000). Together, these findings suggest that accurately capturing the full spectrum of language abilities using parent-reported measures with a small number of items may be problematic.

The strongest agreement between child language measures was for the Language for Learning cohort. This may reflect the study sample of slow-to-talk toddlers, as well as the use of a standardized language assessment for this cohort, compared with the videotaped observational measure used in the Early Home Learning Study cohort. Some disagreement between measures may be attributable to differences in the constructs captured using each measure. While the SSLM, Communicative Development Inventory, and Preschool Language Scale specifically measure children’s expressive language, the Early Communication Indicator and Ages and Stages Questionnaire include some aspects of non-verbal communication. For example, the Early Communication Indicator includes the frequency of a child’s communicative gestures, as well as vocalizations, single words and multiple words. The Ages and Stages communication subscales include items which measure both expressive and receptive language. The RMA plots provided a clear means of identifying the presence of proportional bias; the six child language plots showed minimal proportional bias, suggesting that any bias between the measures was relatively consistent across the distribution of scores.

The strongest predictor of the difference between language measures was child age; however, the direction of this association varied for each cohort. Parents of older children in the Language for Learning cohort tended to report higher scores on parent-reported measures, whereas parents of older children in the Early Home Learning Study cohort tended to report higher scores on the direct measure. Previous research has shown that parents’ ability to accurately report on their child’s language development may deteriorate as children grow older and their vocabulary expands and language use becomes more complex (Law and Roy, 2008). Differences between these cohorts may also be attributable to the child age ranges (24 months and 6–36 months, respectively), as well as the nature of the selected measures. For example, parents of children aged less than 18 months participating in the Early Home Learning Study were asked about receptive as well as expressive vocabulary. In addition, the Early Communication Indicator only assessed *observable* features, such as gestures, vocalizations, single and multiple words. Regardless, it is remarkable that child age was such a highly significant predictor for the Language for Learning cohort, given the narrow range of child ages (*M* = 24.4 months; *SD* = 1.1 months). At this young age, language develops rapidly and a small amount of time can result in quite different language scores. This finding highlights the complexity of measuring language in young children, as well as the importance of selecting measures specific to child age in years and months.

Temperament also emerged as a predictor of child language difference scores, particularly for the Early Home Learning Study cohort. Perhaps surprisingly, more difficult child temperament was generally associated with less discrepancy between language measures. This may be due to parents of children with challenging behaviors having greater awareness of their child’s behavior and development, permitting greater accuracy in parent-reported measures. Again, this could be more apparent through parents’ use of social comparison with the child’s peers. It is also possible that children with behavioral difficulties are the children with poorer language abilities, for whom the strongest agreement was evident. Indeed, there is evidence that language and behavioral difficulties can occur comorbidly (Carpenter and Drabick, 2011). The nature of the assessment – structured assessment or videotaped observation – as well as the presence of the researcher in the home, may also contribute to differences between measures of children’s expressive language.

### Parenting Behaviors

Slightly poorer agreement was observed between measures of parenting behaviors compared to the language measures. We found relatively strong agreement between the parent-reported Home Activities with Child and the Indicator of Parent–Child Interactions Positive Caregiver Score, compared with the parent-reported Parental Verbal Responsivity and the IPCI. As a four-item measure, the PVR performed more poorly as an indicator of parental responsiveness, whereby a ceiling effect led to restricted variation in scores. This measure also showed low internal consistency, making it a less reliable measure. Both the PVR and HAC showed a tendency to underestimate parental responsiveness at the lower and upper extremes. Overall, our findings suggest that a brief parent-reported measure of the frequency of engagement in parent-child activities in the home (HAC) may represent a reliable indicator of parental responsiveness and engagement, which shows relatively good agreement with a comprehensive observational measure. For studies with limited resources, the HAC could be a feasible alternative to time-intensive and costly observation required for the IPCI. It should be acknowledged that some disagreement between the measures of parenting behaviors could be explained by differences in the construct being measured or coded. For example, the PVR measures parents’ *verbal* responsiveness specifically, whereas the HAC assesses parent engagement and responsiveness more broadly, including both verbal and non-verbal behaviors. Both the PVR and HAC ask parents about the frequency with which they engage in everyday activities, such as reading books or talking about the day during mealtimes. The Positive Caregiver Total score derived from the IPCI captured the frequency of both verbal and non-verbal parenting behaviors, such as using descriptive language, and following the child’s lead (i.e., quantity and quality of parenting behaviors).

Language other than English was the strongest explanatory factor of the difference between parent-reported and directly measured parenting behaviors. Families with a non-English speaking background tended to report lower scores on the directly measured videotaped observation, the IPCI, and higher scores on both the PVR and HAC. This may be attributable to potential acquiescence (i.e., consistently indicating positive responses). Acquiescence has been shown to vary cross-culturally, for example, strong cultural preferences to avoid uncertainty can lead to a tendency to select more extreme values (Smith, 2004). Findings may also reflect cultural differences in the frequency with which parents and children engage in the activities being measured (e.g., HAC: “telling stories to your child” or PVR: “playing peek-a-boo or hide-and-seek”). It is also possible that parents and children with a non-English speaking background felt less comfortable than native English speakers during the videotaped activities. Furthermore, these differences could be attributable to difficulties understanding the verbal instructions of the videotaped activities, or difficulties in coding parent utterances during these activities. Lastly, we acknowledge that parents’ English proficiency may vary to that of the child, particularly in early childhood when children have not yet been exposed to English in the school environment.

The small proportion of variance explained by the socio-demographic factors for parenting behaviors suggests that other unmeasured factors may be responsible for differences between these measures. The current study was limited by the data collected in the two datasets analyzed; it is possible that other factors may have greater explanatory power than variables assessed in these studies. For example, the parent or child’s unique and subjective experience of the assessments, understanding of the task requirements or the questionnaire items, cultural factors affecting parent–child interactions, discomfort during the assessment, rapport with the assessor, experiences of fatigue or illness at the time of the assessment or external factors causing stress or distraction may have been more relevant predictors of agreement. Quantile regression analyses revealed that the associations between the socio-demographic factors and the difference scores remained stable across the quantiles of agreement. The only exception was the comparison between the parent-reported HAC and the directly measured videotaped observation, the IPCI. Greater discrepancy between these measures was associated with parents with a lower income. The five HAC items refer to everyday parent–child activities; however, many of these activities require resources such as books and toys, which may be less readily available for parents who have a very low income. Indeed, this link between families of a lower socio-economic status and the provision of a less stimulating home environment is well-established (e.g., Davis-Kean, 2005).

### Implications

This study provides evidence to guide the selection of appropriate measures for parents and their children aged 6–36 months. Method comparisons such as this are critical for supporting the collection of high data quality and the appropriate allocation of limited resources. Our data suggest that brief parent-reported measures of child language may be used with reasonable confidence for children up to 3 years of age. Particularly for children who are slow-to-talk, parent-reported measures may provide an accurate and cost-effective means of monitoring development over time. Findings indicate that agreement between measures of parenting behaviors is generally poorer than child language measures. Parenting behaviors can be difficult to accurately measure, given that social desirability can cause parents to consciously or unconsciously change the way they respond on parent-reported questionnaires (Zaslow et al., 2006; Law and Roy, 2008), or the way they behave during observations (Arney, 2004). It is also conceivable that parents are more able to objectively report on their child’s language but are less objective when evaluating their own behaviors (e.g., parenting responsiveness). Despite this, the parent-reported Home Activities with Child measure showed relatively strong agreement with the direct videotaped observation, the Indicator of Parent–Child Interactions, with minimal proportional bias. This suggests that measuring the frequency of developmentally beneficial activities such as reading, story-telling, singing, or involving the child in everyday tasks at home, provides a valid indication of parents’ general level of engagement and responsiveness.

When selecting measures, it is important to consider the purpose for which the data is being generated; a brief parent-reported measure of children’s expressive language or communicative development such as the SSLM, Communicative Development Inventory or Ages and Stages Questionnaire may be sufficient for large-scale studies where time and resources are limited and a large pool of data is required. Whereas a clinician making decisions about treatment options for a young child may be best to draw on both direct and parent-reported measures to ensure a comprehensive assessment.

The study has significant implications for the analysis of method comparisons. We demonstrate how the Bland–Altman Method and RMA regression permit a comprehensive assessment of agreement across the distribution of scores. While correlational analyses reported here were comparable to those reported elsewhere for similar constructs, analyses using the Bland–Altman Method and RMA regression clearly show how correlations have the potential to be misleading. Correlations represent a single figure which summarizes association across the spectrum of scores, whereas agreement may vary between higher and lower scores. The level of detail generated by these more comprehensive techniques is crucial for identifying groups of children or parents for whom one method may be sufficient (in the case of strong agreement), or for whom multiple methods or an agreed “gold standard” measure may be necessary (in the case of poor agreement).

### Strengths and Limitations

To our knowledge, this is the first study to apply the Bland–Altman Method to a comparison of parent-reported and directly measured behaviors. This technique permitted the identification of patterns of bias across the distribution of scores. As a result, we were able to identify groups of children or parents for whom multi-method administration may be necessary, or for whom one method of measurement may be permissible. Rarely used in non-medical fields, the Bland–Altman method represents a relatively simple and visually appealing technique. The approach lends itself to other comparisons such as parent-, teacher-, and child-report of the same questionnaire (e.g., Gabbe et al., 2010; Stolarova et al., 2014), or comparisons of the same measure across time points (e.g., Eadie et al., 2014). Another strength is the use of RMA regression to identify the magnitude of proportional bias between methods. Together, Bland–Altman and RMA regression plots represent powerful visuals for comparing measures which can be executed and interpreted with relative ease. The use of quantile regression analyses also allowed us to determine whether associations between socio-demographic factors and agreement varied across quantiles of agreement, which is not possible using standard ordinary least squares regression.

We acknowledge that we were limited to the measures available within existing datasets, and therefore cannot presume agreement findings are generalizable to other measures of child language and parenting behaviors. Despite this, our measures are commonly used and well-validated. It should be noted that the PLS-4 was only normed on US data at the time of data collection; no Australian norms were available. Data also pertained to a sample of toddlers identified as “slow-to-talk” at age 18 months, and another sample of families experiencing social disadvantage; different populations may yield different results. We also recognize that each of the measures used in this study will, naturally, capture slightly different aspects of child language or parental responsiveness. As with any method comparison, total agreement is not expected, nor is it feasible to strive for this; some degree of measurement error is inevitable (Bland and Altman, 1999). Regardless, method comparisons are critical for determining whether measures are potentially interchangeable, and may contribute to more effective allocation of limited resources and strengthened data quality.

### Future Research

We suggest that researchers consider applying these techniques to method comparisons of other commonly used early childhood language measures, such as Clinical Evaluation of Language Fundamentals (CELF), and with larger sample sizes where possible to ensure greater precision around the limits of agreement. Our future research will employ qualitative methodologies to determine how parents’ unique and subjective experiences of assessments may further explain and contextualize agreement findings. This is particularly important given that a broad range of socio-demographic factors explained little variability in the difference scores for a number of measures. It is possible that parents and children vary in their level of comfort when behaviors are being measured directly (i.e., videotaped observations or standardized assessments), especially for participants who are not native English speakers. Exploring this qualitatively could go some way to understanding agreement and supporting data collection methods which optimize the validity of parent and child data.

## Conclusion

This study demonstrates how well-established statistical techniques from non-psychology disciplines can be applied to method comparisons in the field of psychology. The Bland–Altman Method is a useful visual technique for detecting bias and for determining potential interchangeability between measurement methods, which can be used in combination with RMA regression to identify the presence of both fixed and proportional bias. Although we found correlations which were consistent with previous comparisons of child language and parenting behaviors, agreement varied substantially across the distribution of scores, demonstrating the need for these more comprehensive techniques. On the whole, poorer agreement was observed for children with average expressive language abilities, and stronger agreement was observed for children with very poor or more advanced language abilities. Slightly poorer agreement was observed between measures of parenting behaviors, with the weakest agreement seen for parents of average responsiveness. As would be expected, stronger agreement was observed between comparisons of two parent-reported measures. Further research is required to determine agreement between other commonly used measures and how the participant experience may explain agreement between parent-reported and directly measured behaviors. We recommend that journal editors encourage the use of the Bland–Altman Method and RMA regression techniques and discourage the use of correlations for method comparisons.

## Author Contributions

SB was responsible for leading the preparation of this manuscript. SB, FM, EW, NH, and SR all made substantial contributions to the study design, analysis, interpretation, writing and revision of the manuscript. All authors provided approval for publication.

## Conflict of Interest Statement

The authors declare that the research was conducted in the absence of any commercial or financial relationships that could be construed as a potential conflict of interest.
