# Supplementary material for: The Agreement between Parent-Reported and Directly Measured Child Language and Parenting Behaviors
Source: Front Psychol. 2016 Nov 11;7:1710. doi: 10.3389/fpsyg.2016.01710 (PMC5104739; doi:10.3389/fpsyg.2016.01710)
Supplement: Supplementary file 1 [file Table_1.docx]

Supplementary Material

**Agreement between parent-reported and directly measured child language and parenting behaviors**

**Bennetts, S.K*, Mensah, F.K., Westrupp, E.M., Hackworth, N.J.., & Reilly, S.**

*** Correspondence:** Shannon Bennetts: [shannon.bennetts@mcri.edu.au](mailto:shannon.bennetts@mcri.edu.au)

# Supplementary Tables

**Table 8.** Unadjusted associations for Language for Learning difference scores and sociodemographic factors.

|  | ASQ vs. PLS-E | | | | SSLM vs. PLS-E | | | ASQ vs. SSLM | | |
| --- | --- | --- | --- | --- | --- | --- | --- | --- | --- | --- |
|  | Coeff. | *p* | | 95% CI | Coeff. | *p* | 95% CI | Coeff. | *p* | 95% CI |
| Parent age (years) | .01 | .69 | -.02, .03 | | -.02 | .16 | -.05, .01 | .03 | .03 | .00, .05 |
| Child age (months) | .23 | <.001 | .12, .35 | | .33 | <.001 | .22, .44 | -.11 | .04 | -.21, -.01 |
| Child gender (female) | .16 | .21 | -.09, .41 | | .25 | .06 | -.01, .50 | -.08 | .48 | -.30, .14 |
| Single parent | .20 | .47 | -.34, .74 | | .51 | .10 | -.10, 1.12 | -.39 | .14 | -.91, .13 |
| Household unemployment | -.25 | .39 | -.82, .32 | | .20 | .54 | -.45, .86 | -.48 | .09 | -1.03, .07 |
| No higher education | .01 | .93 | -.24, .26 | | .20 | .14 | -.06, .46 | -.16 | .16 | -.38, .06 |
| Income |  |  |  | |  |  |  |  |  |  |
| low vs. mid | .01 | .96 | -.35, .36 | | -.27 | .16 | -.64, .11 | .27 | .09 | -.05, .58 |
| low vs. high | .09 | .60 | -.25, .43 | | -.35 | .06 | -.71, .01 | .43 | <.01 | .13, .74 |
| SEIFA/100(less disadvantage) | -.21 | .08 | -.44, .02 | | -.26 | .04 | -.51, -.01 | -.01 | .90 | -.22, .20 |
| LOTE | .14 | .50 | -.28, .57 | | -.18 | .42 | -.63, .26 | .32 | .10 | -.06, .70 |
| Difficult child temperament | -.09 | .30 | -.27, .08 | | .13 | .16 | -.05, .31 | -.21 | <.01 | -.36, -.06 |

LOTE=Language other than English.; ASQ=Ages & Stages Questionnaire, communication subscale; PLS-E=Preschool Language Scale, expressive language score; SSLM=Sure Start Language Measure.
