# Supplementary material for: The Agreement between Parent-Reported and Directly Measured Child Language and Parenting Behaviors
Source: Front Psychol. 2016 Nov 11;7:1710. doi: 10.3389/fpsyg.2016.01710 (PMC5104739; doi:10.3389/fpsyg.2016.01710)
Supplement: Supplementary file 2 [file Table_2.docx]

Supplementary Material

**Agreement between parent-reported and directly measured child language and parenting behaviors**

**Bennetts, S.K*, Mensah, F.K., Westrupp, E.M., Hackworth, N.J.., & Reilly, S.**

*** Correspondence:** Shannon Bennetts: [shannon.bennetts@mcri.edu.au](mailto:shannon.bennetts@mcri.edu.au)

**Table 9.** Unadjusted analysis for the Early Home Learning Study difference scores and sociodemographic factors (child language measures).

|  | ASQ vs. ECI | | | ASQ vs CDI | | | CDI vs ECI | | |
| --- | --- | --- | --- | --- | --- | --- | --- | --- | --- |
|  | Coeff. | *p* | 95% CI | Coeff. | *p* | 95% CI | Coeff. | *p* | 95% CI |
| Parent age (years) | .00 | 1.0 | -.05, .05 | -.01 | .39 | -.04, .02 | -.01 | .74 | -.05, .04 |
| Child age (months) | -.11 | <.001 | -.13, -.08 | -.01 | .18 | -.03, .00 | -.08 | <.001 | -.11, -.06 |
| Child gender (female) | .43 | .09 | -.07, .94 | -.08 | .58 | -.37, .21 | .35 | .14 | -.12, .81 |
| Single parent | .43 | .32 | -.42, 1.28 | .33 | .22 | -.20, .86 | .12 | .76 | -.65, .89 |
| Household unemployment | .57 | .17 | -.24, 1.38 | .35 | .19 | -.17, .86 | .25 | .50 | -.49, .99 |
| No higher education | -.11 | .69 | -.61, .41 | -.20 | .17 | -.49, .09 | .10 | .67 | -.37, .57 |
| Income  low vs mid  low vs high | .22  .06 | .65  .88 | -.75, 1.20  -.73, .85 | -.34  .04 | .23  .88 | -.89, .21  -.41, .48 | .12  .15 | .79  .68 | -.77, 1.01  -.58, .88 |
| SEIFA/100 (Less disadvantage ) | -.28 | .24 | -.75, .19 | -.08 | .56 | -.33, .18 | -.13 | .57 | -.56, .31 |
| LOTE | .13 | .64 | -.43, .70 | .31 | .09 | -.04, .66 | -.21 | .44 | -.73, .32 |
| Difficult child temperament | -1.17 | <.001 | -1.67, -.67 | -.15 | .39 | -.50, .20 | -.97 | <.001 | -1.43, -.51 |
| High parenting self-efficacy | .30 | .04 | .01, .60 | .06 | .46 | -.10, .22 | .22 | .11 | -.05, .48 |
| Poor health-related quality of life | -.27 | .06 | -.55, .01 | -.03 | .70 | -.18, .12 | -.23 | .07 | -.48, .02 |
| Greater psychological distress | -.04 | .38 | -.12, .05 | -.01 | .59 | -.06, .03 | -.04 | .32 | -.11, .04 |

LOTE=Language other than English; ASQ=Ages & Stages Questionnaire, communication subscale; ECI=Early Communication Indicator; CDI=Communicative Development Inventory
