# Supplementary material for: The Agreement between Parent-Reported and Directly Measured Child Language and Parenting Behaviors
Source: Front Psychol. 2016 Nov 11;7:1710. doi: 10.3389/fpsyg.2016.01710 (PMC5104739; doi:10.3389/fpsyg.2016.01710)
Supplement: Supplementary file 3 [file Table_3.docx]

Supplementary Material

**Agreement between parent-reported and directly measured child language and parenting behaviors**

**Bennetts, S.K*, Mensah, F.K., Westrupp, E.M., Hackworth, N.J.., & Reilly, S.**

*** Correspondence:** Shannon Bennetts: [shannon.bennetts@mcri.edu.au](mailto:shannon.bennetts@mcri.edu.au)

**Table 10.** Unadjusted analysis for the Early Home Learning Study difference scores and sociodemographic factors (parenting behavior measures).

|  | PVR vs IPCI | | | HAC vs IPCI | | | PVR vs HAC | | |
| --- | --- | --- | --- | --- | --- | --- | --- | --- | --- |
|  | Coeff. | *p* | 95% CI | Coeff. | *p* | 95% CI | Coeff. | *p* | 95% CI |
| Parent age (years) | -.04 | .05 | -.09, .00 | -.03 | .10 | -.07, .00 | -.02 | .14 | -.05, .00 |
| Child age (months) | -.02 | .08 | -.04, .00 | .00 | .88 | -.02, .02 | -.02 | <.01 | -.04, -.01 |
| Child gender (female) | -.02 | .94 | -.46, .43 | -.47 | .02 | -.88, -.07 | .20 | .16 | -.08, .48 |
| Single parent | -.17 | .67 | -.93, .60 | -.30 | .41 | -1.01, .41 | .03 | .92 | -.50, .55 |
| Household unemployment | -.29 | .48 | -1.10, .52 | -.18 | .65 | -.94, 58 | -.13 | .63 | -.64, .39 |
| No higher education | .41 | .07 | -.03, .84 | .29 | .17 | -.12, .70 | -.03 | .83 | -.31, .25 |
| Income  low vs mid  low vs high | .27  .13 | .53  .70 | -.57, 1.10  -.53, .79 | .08  .20 | .84  .53 | -.71, .87  -.42, .82 | .20  .04 | .45  .87 | -.33, .74  -.40, .48 |
| SEIFA/100 (Less disadvantage) | -.28 | .15 | -.67, .10 | -.04 | .83 | -.40, .32 | -.13 | .28 | -.38, .11 |
| LOTE | .97 | <.01 | .37, 1.56 | 1.02 | <.001 | .47, 1.57 | .00 | .98 | -.35, .34 |
| Difficult child temperament | -.55 | .04 | -1.07, -.04 | -.14 | .58 | -.62, .35 | -.30 | .09 | -.64, .04 |
| High parenting self-efficacy | .29 | .02 | .05, .54 | .09 | .43 | -.14, .33 | .11 | .19 | -.05, .26 |
| Poor health-related quality of life | -.07 | .54 | -.31, .16 | .01 | .95 | -.21, .23 | -.04 | .63 | -.19, .11 |
| Greater psychological distress | .03 | .45 | -.05, .10 | .01 | .71 | -.06, .08 | .04 | .11 | -.01, .08 |

LOTE=Language other than English; PVR=Parental Verbal Responsivity scale; IPCI=Indicator of Parent-Child Interactions, positive caregiver score; HAC=Home Activities with Child scale.
